# Are Women Accepted at Work? Gender Discrimination in Chinese Firms

**DOI:** 10.1111/cars.70043

**Published:** 2026-06-05

**Authors:** Anson Au

**Affiliations:** ^1^ Department of Applied Social Sciences The Hong Kong Polytechnic University Hung Hom Kowloon People's Republic of China

**Keywords:** discrimination, private firms, state firms, women's employment

## Abstract

To what extent is women's employment accepted in modern Chinese firms? Drawing on pooled 2017 and 2021 nationally representative Chinese General Social Survey (CGSS) data, this article estimates the socioeconomic predictors and prevalence of the belief that women should be stay‐at‐home wives instead of working in Chinese firms. The results illustrate that women in Chinese firms have socioeconomic resources commensurate with men, but they remain targeted by the persistent patriarchal belief that women should marry and be relegated to the household instead of work. Contrasting against conventional accounts of private firms as anti‐discriminatory, private firm workers are observed to believe that women should be stay‐at‐home wives instead of working more than state firm workers. In particular, women workers are more likely to resist the traditional belief that women should be stay‐at‐home wives instead of working, whereas men appear more likely to endorse this belief, especially in private firms. The results suggest that women are not simply passive recipients of patriarchal beliefs, but exhibit a willingness to challenge their symbolic boundaries and reject traditional expectations of unequal gender relations in the workplace.

## Résumé

Dans quelle mesure l'emploi des femmes est‐il accepté dans les entreprises chinoises modernes? S'appuyant sur des données regroupées de l'Enquête sociale générale chinoise (CGSS) de 2017 et 2021, représentatives à l'échelle nationale, cet article évalue les facteurs socio‐économiques prédictifs et la prévalence de la croyance selon laquelle les femmes devraient rester au foyer plutôt que de travailler dans les entreprises chinoises. Les résultats montrent que les femmes dans les entreprises chinoises disposent de ressources socio‐économiques comparables à celles des hommes, mais qu'elles restent la cible d'une croyance patriarcale persistante selon laquelle les femmes devraient se marier et se consacrer aux tâches ménagères plutôt que de travailler. Contrairement aux descriptions conventionnelles des entreprises privées comme étant non discriminatoires, on observe que les employés du secteur privé sont plus nombreux que ceux des entreprises publiques à croire que les femmes devraient être des épouses au foyer plutôt que de travailler. En particulier, les travailleuses sont plus susceptibles de s'opposer à la croyance traditionnelle selon laquelle les femmes devraient rester au foyer plutôt que de travailler, tandis que les hommes semblent plus enclins à adhérer à cette croyance, en particulier dans les entreprises privées. Les résultats suggèrent que les femmes ne sont pas simplement des réceptrices passives des croyances patriarcales, mais qu'elles manifestent une volonté de remettre en question leurs limites symboliques et de rejeter les attentes traditionnelles en matière de relations de genre inégales sur le lieu de travail.

## 1 Introduction: Women's Paid and Unpaid Labour

Large bodies of evidence in social stratification, economic sociology, and economics have identified gendered disadvantages that disproportionately affect women in wages, hiring, and performance evaluations (Campero 2021; Correll et al. 2020; Rivera and Tilcsik 2019). The patriarchal gender role beliefs that discriminate against women's employment are motivated by traditional patriarchal values that preclude their participation in paid labour (Charles et al. 2023; Elliott and Reid 2019; Ferree 2020). The exclusion of women from paid employment and their relegation to unpaid labour through marriage has been widely documented as a trade‐off that economically disempowers them. Hochschild (2012) classically argued that women who elect to marry are often expected to undertake a “second shift,” namely, substantially greater amounts of unpaid housework that come at the cost of occupational attainment (see Quadlin and Doan 2018). In addition, married women face severe penalties in income, education, and promotion opportunities (England et al. 2020; Gonalons‐Pons and Gangl 2021; Ludwig and Brüderl 2018). Panel evidence shows that these gendered disadvantages have culminated in married women disproportionately choosing to turn to part‐time work or withdrawing from the workforce altogether (Biemann et al. 2012).

Indeed, despite storied gains in women's participation in paid work over the past ten years in advanced capitalist economies around the world, rates of paid work among married women are consistently out of sync with those of their male counterparts. Cognizant of the gender wage gap (in part because of their married status and greater share of the housework), married women in developed economies have been increasing their hours of paid work in a bid to overcome their handicap (Jones et al. 2015).

Women face disproportionately fewer opportunities for upward mobility on account of patriarchal cultural norms that constrain their place in the household (Seeberg and Luo 2018). As a result, women disproportionately suffer from greater barriers to obtaining basic economic resources (education and income) and pathways to upward mobility (overcoming segregation in the labour market) that result in their being sorted into lower‐status occupations (Wang and Cheng 2021; Wu and Qi 2017). Moreover, it is increasingly apparent that these myriad forms of deprivation among women are affected by attitudes towards women's pathways for upward mobility and beliefs about gender equality within the workplace itself.

As Risman (2004, p.433) argues, there exist “cultural and institutional components” of the social structure of gender that shape the “interactional expectations” held of women's employment. Analytically focusing on gender as such a social structure has been fruitful for excavating patriarchal gender role beliefs about women's employment. Recent work within this scope has yielded insights into how these gendered beliefs are embedded in institutional design and sustained by cultural meanings attached to technical work, such as the masculinist construction of computer programming, hardware management, and engineering skills (Alegria and Branch 2015; Alegria 2019; Alfrey and Twine 2017). Organizational contexts thus emerge as social spaces where such beliefs manifest and create knock‐on effects on women's income and prospects of upward mobility (Smith‐Doerr et al. 2019), becoming one of the most consequential sites for the contemporary reproduction of gender inequality.

In formal organizational contexts, women are consistently evaluated lower than men for comparable levels of performance, resulting in less credit for the successes they achieve (Heilman et al. 2019; Manzi and Heilman 2021). Even with comparable amounts of technical skills as their male counterparts, women are implicitly held to unwritten standards such as how affable they appear to their male colleagues (Lee and Huang 2018). Women who stray from such informal expectations are found to be punished in official performance assessments (Correll et al. 2020), enabling implicit beliefs about patriarchal gender roles to influence formal evaluations of women's ability to be hired or promoted (Campero 2021; Dobbin and Kalev 2021; Rivera and Tilcsik 2019).

Recent research on perceived group threat and exclusion demonstrates that individuals who perceive status erosion or competitive pressure may respond by endorsing more exclusionary norms (Borinca et al. 2025). These dynamics help explain why men in rapidly modernizing labour markets may support traditionally patriarchal gender role beliefs, not solely due to cultural persistence but as a reaction to perceived competition from women.

In conversation with this literature, this article extends this work to the context of Chinese firms. As the world's largest national economy by the Organization for Economic Co‐operation and Development's (OECD, 2023) GDP estimates (a GDP of US$30.09 trillion as of 2022, ahead of the U.S.’ $25.46 trillion), China constitutes a salient empirical case with which to examine gendered beliefs in contemporary market economies. Despite becoming a major driver of global economic growth and the largest trading partner for many liberal democracies with advanced capitalist economies, China experiences a unique dislocation between its advanced economic development and patriarchal beliefs against women, reporting nearly double the amount of organizational gender discrimination as the OECD average (OECD 2022). Unlike Canada or other OECD countries, where public‐sector equity legislation constrains discriminatory practices, China's state‐sector governance ties employment stability to political legitimacy. This creates distinct organizational incentives: state firms may reproduce older values through stable workforces, whereas private firms may generate new tensions due to rapid turnover and competitive pressures.

This article foregrounds the role of organizational contexts as places of employment in shaping patriarchal beliefs about women across prominent types of firms in China, namely, state and private firms. Should women marry and relegate themselves to the household, as conventional patriarchal beliefs would hold, or should women be permitted to work? Analyzing nationally representative microdata in China, this article examines variations in these beliefs among state and private firms and the root socioeconomic causes underpinning them across intersections of gender and firm type.

## 2 Theorizing Patriarchal Beliefs About Women's Employment

Classic lines of sociological research have suggested cognitive attachments to social scripts about patriarchal gender roles as a driving force behind intolerance against women in the workplace. In *Distinction*, Bourdieu (1984) seminally posited that class conditions imply “social conditioning” (p.101). This conditioning, like a *habitus*, drives members of a profession to enact “overtly or implicitly guiding co‐optation choices” based on “age, sex, social or ethnic origin” that gatekeep “entry into the profession and right through a career, so that members of the corps who lack these traits are excluded to marginalized… [especially] women” (p.105).

Although patriarchal beliefs are learned as scripts, they are at their core selectively activated, redefined, and may even be overlooked by individuals to obtain their desired outcomes (Swidler, 2003). Indeed, these beliefs (as scripts) are themselves inherited from individuals’ social environment as “a set of heuristics, hunches and shallow (but useful because they work most of the time) practical skills that allow persons to best interface externalized structures, contexts and institutions” (Lizardo and Strand 2010, p.206). Put differently, patriarchal beliefs, like all scripts, tell individuals of possible courses of action or thought to achieve their desired ends, but do not decide these ends themselves nor do they offer rationales for such ends (Kaufman 2004, p.340; Vaisey 2008; Vaisey and Miles 2014).

In China, these scripts become the basis of two polarized pathways for women: to marry and be relegated to the household, or to enter the workplace. For women in China, marriage has been widely seen as a rite of passage into adulthood throughout Chinese history, in part because of social dependence on the family as a source of welfare in a poor country committed to modernizing without foreign assistance (Wolf 1984). In the throes of the civil war that preceded the foundation of the modern Chinese state, Mao (1953) observed in his *1927 Report*:

A man in China is usually subjected to the domination of three systems of authority: (1) the system of the state (political authority)… (2) the system of the clan (clan authority)… and (3) the systems of gods and spirits (theocratic authority)… as to women, apart from being dominated by the three systems above, they are further dominated by men (the authority of the husband). These four kinds of authority… represent the whole ideology and institution of feudalism and patriarchy, and are the four enormous cords that have bound the Chinese people. (1953, p.40)

These “four cords” were predicated on marriage between a man and a woman, within which there existed a clearly defined hierarchy that not only subordinated women but also bound them to the household as a perceived source of sustenance for the family unit through their domestic labour and obedience to their husbands. Charged with a sense of moral worthiness and ordained by the other cords of authority, in all their ancestral gods and clan traditions, the bondage of women to the household through marriage was pseudo‐divine and historically institutionalized.

It was implied that men would be incapable of performing paid labour and children would not be raised sufficiently without this genre of unpaid domestic labour by women in the household (Liu 2004). So powerful was this perception that statehood was contingent on the role of women in marriage, even subsequent attempts by the Chinese state to reform gender relations and liberate women to work were met with quiet resistance and later abandonment (Palmer 1995).

The historical significance of marriage for women casts a long shadow on their life chances in the nation today, casting their life chances into domestic labour and paid labour as the only two, polarized possible outcomes. Recent 2022 data on national employment from the World Bank (2022), for instance, show that the paid labour force participation rate among women in China stands at 61.1%, notably lower than their male counterparts, who report a 72.6% participation rate. Data from sub‐national administrative regions within China said to be more liberal, such as Hong Kong, reveal a similar discrepancy between men and women, where the labour participation rate stands at 64.7% for men, but 52.9% for women (Census and Statistics Department of Hong Kong 2023).

Moreover, follow‐up survey questions inquiring into reasons for women's economic inactivity (their low labour participation rate) found that a significant proportion of women (35.2% of all women) cited engagement with household duties as the reason for their inability to obtain a job, whereas men's reasons for economic activity were linked to attendance at educational institutions. The prospects of marriage (entry into unpaid domestic labour) and employment (entry into paid labour), therefore, represent separate and polarized pathways for women's life course.

It bears noting that women may also partake in gender discrimination, especially given the institutionalization of these patriarchal norms in the “four cords” of modern China. This work gains credence from and contributes to the literature on conflict against minorities by majority groups when the latter feel a sense of threat (see Bansak et al. 2016; De Coninck 2020; Olson et al. 2007). Where much of this work has focused on racial and ethnic relations, the present article extends this dialogue to examine how men (and women) may react against women occupying paid roles in the workplace, rather than unpaid roles in the household.

### 2.1 Boundaries, Group Threat, and Patriarchal Beliefs About Women's Employment

Group threat theories posit that responses to perceived exclusion shape gender attitudes in the workplace, such that when people perceive their group as marginalized or threatened, they report stronger in‐group preferences and greater support for exclusionary norms (Borinca et al. 2025; Emerson and Murphy 2015; Von Hippel et al. 2011). While this work is prominently couched in psychology, it aligns with sociological research that identifies the role of boundaries in the workplace to affect inequality (Lamont & Molnar 2002). Lamont and Fournier (1992) assert that symbolic boundaries are often erected in the workplace based on ascribed characteristics, including gender. These boundaries need not be spatial, but assume the form of words and categories that label women and render them socially distant (p.11).

So codified are these boundary distinctions that, as Epstein (2007) asserts, women are often forced to reassure their male counterparts in the workplace of their respect for traditional gender roles and commitment to family over work. These boundaries place at risk women's opportunities to accomplish job tasks, earn promotions, and retain job security, because they are reinforced when “repeatedly tested by people who are on the fringes of the group and repeatedly defended by those within it” (Stein 2001, p.8). These actions, found to be performed by women even across class strata, recursively construct and reinforce their disadvantaged social position as the status quo (Yodanis 2002, p.326).

In a similar fashion, such boundaries animate tension and conflict between men and women in Chinese firms. Within Chinese private firms, where promotion systems are competitive and evaluation criteria fluid, men work to erect and retain the symbolic boundaries dividing men and women, preserve the status quo, and ultimately enact social distance against women (Au 2024a; Shen and Wang 2023; Yang and Li 2009). Au (2024a) finds that when faced with the prospect of a recession, for instance, male managers prefer to lay off women first in Chinese firms; an evocative example of men turning symbolic boundaries into spatial ones by evicting women from the shared space of the workplace.

The associations of job tasks and job roles with masculinity and femininity lead to a market‐wide segregation of men and women into different jobs. Kmec et al. (2010), for instance, find that job informants tend to make unsolicited job referrals (“nonsearching”) for their contacts based on gender stereotypicality: men and women contacts are segregated into “male‐typical” and “female‐typical” job referrals, whereas the women who defy this stereotypicality and work in male‐dominated fields are informally penalized with lower job rewards compared to fellow men. This penalty is explained by the fact that workers, who rely on gender stereotypicality to queue jobs and firms (Reskin and Roos 1990), police gendered symbolic boundaries about job fits and stigmatize transgressions across gendered lines.

This preoccupation with ordering jobs by gender is accentuated in the Chinese labour market, where women's entry into paid labour has been a relatively recent phenomenon and relegation to the household has instead been their traditional life course (Wolf 1984), compared to other societies with a longer history of women in the workplace (Aksu et al. 2025). Moreover, Chinese firms possess a stagnant employee pool and few layoffs over decades (Au 2024a), that amplifies the symbolic boundaries erected by the predominantly male groups in power (Lamont & Fournier 1992).

This article examines how socioeconomic resources might dampen the likelihood that these symbolic boundaries are invoked by men to penalize women. Socioeconomic resources are defined as income, education, job precarity, and job control. Taken to represent the financial security and social capital sufficient to secure their present job and improve job search outcomes (Lin 2001), socioeconomic resources offer a proxy for the perceived threat that incoming women workers pose to the job security of existing workers. Having income provides a sufficient sense of stability that enables individuals to be tolerant of newcomers and other groups (Quillian 1996; Rosenstein 2008). In racially homogeneous work settings like China, dominant group members whose individual interests (economic or otherwise) are not directly threatened by an opposing group are less likely to express prejudice (Quillian 1996).

These connections between socioeconomic resources and patriarchal beliefs about women's employment are especially prevalent in China, given that socioeconomic resources are often taken as positional cues (status signals) that contacts refer to when deciding whether to offer job referrals and social support (Burt 2019; DiTomaso and Bian 2018). Cross‐national studies have identified similar links between material well‐being and patriarchal beliefs about women's employment. In a cross‐national study of beliefs in equality for men and women in Europe and the U.S., Olson et al. (2007) observed that women were encouraged (discouraged) to enter the workplace when the national economy was growing (recessionary). The ability of women to obtain equality in the workplace was, therefore, contingent on the broader economic resources that they and their evaluators possessed. Pepin and Cotter (2018) echo these results by identifying rising support for gender egalitarianism from 1976 through to the end of the twentieth century, coincident with broader patterns of rising education, employment, and income.

Accordingly, this article hypothesizes that:

Hypothesis 1 Greater socioeconomic resources predict a lower likelihood of possessing the belief that women should be stay‐at‐home wives instead of working.

### 2.2 Firm Effects and Patriarchal Beliefs About Women's Employment

This article further examines the organizational context in which patriarchal beliefs are exercised, namely, the type of firm in which individuals are employed. In organizations, firm structures constitute seedbeds for conditioning individual perceptions of competition that result in a sense of threat and possible hostility against newcomers. Sociological research on gender stereotypes finds that when the existing pool of workers perceives their status as challenged by the inclusion of subordinate groups, they may respond by endorsing exclusionary beliefs that legitimize existing hierarchies (Correll et al. 2007; Gorman 2005). The gender‐stereotypicality of hiring preferences is deeply institutionalized, according to Gorman (2005), who observes that selection criteria are often associated with stereotypically masculine or feminine qualities, which results in a greater proportion of same‐gender hires. Ultimately, this reinforces gender inequality in firms, especially among entry‐level hires, where male decision makers prefer to hire fellow men and fill fewer vacancies with women compared to female decision makers.

Within this scope, the type of firm affects experiences of discrimination in a workplace. Different firm types are theorized to embody different sets of organizational objectives, values, and behaviours that come to influence a firm's decisions on how to allocate financial and human capital on the ground (Fligstein and Goldstein 2022). Embedded in these ideal types of firms, therefore, are powerful paradigms that orient the sense of group threat and patriarchal beliefs about women's employment, among all other facets of employee experiences.

Focused on China, this study foregrounds the distinctions between state and private firms as two of the most prominent ideal types of firms that account for the largest proportion of corporate asset ownership and employee hiring nationwide. Much of the Chinese economy began with state‐owned enterprises (SOEs) and collectively‐owned enterprises (COEs), defined by majority ownership by the government. From the modern foundation of China in 1949, these state firms were responsible for dispensing much of the goods and services consumed by citizens under a system of fixed prices and fixed quotas. From 1978 onward, however, then‐leader Deng Xiaoping implemented the “Open‐Door Policy” that consisted of a set of market‐oriented reforms that radically reorganized firms nationwide. As markets liberalized to replace quota‐driven production with demand‐driven production, state firms were forced to compete with a growing crop of private firms.

As a result, state firms have steadily declined in number compared to private firms, which account for about 96% of all firms (27.545 million out of 28.665 million firms in China) and are a major source of employment nationwide (National Bureau of Statistics of China 2022). State firms have been relegated to managing key sectors of the economy responsible for essential goods and services. Oil and petroleum production in the entire nation, for instance, is monopolized by three SOEs alone (Sinopec, PetroChina, and China National Offshore Oil Corporation), while the natural gas distribution thereafter to cities for energy is coordinated by a handful of firms dependent on state contracts with these three SOEs. For their control over lucrative but smaller parts of the economy, state firms controlled over half of corporate assets nationwide in 2022, an amount that grew by about 10% annually since 1997 (Wang 2022).

This history of the two firm types in Chinese markets imputes different attitudes toward (women's) employment in each. In state firms, state‐mandated constraints on profits reflect a firm logic that treats state firm assets as public goods, rather than private ones for profit. For this reason, even though preserving employee employment and well‐being is a capital expenditure, they are considered a proxy for the stability of the Chinese government and so remain resilient even during economic recessions (Guo et al. 2017). State firms in China are much less likely to resort to layoffs to bolster profits during downturns, barring the prospect of severe economic depressions like the 1997 Financial Crisis (Wang and Luo 2019). Given that layoffs are rare in state firms, the pool of workers in each firm is largely stable.

In other words, state firms may exhibit stronger patriarchal beliefs because their unchanging, aging labour pool that possesses them is resistant to demographic change across decades. This is augmented by a sense of threat that arises when faced with the prospect of new women workers who might disrupt the existing pool of predominantly male workers (Lamont & Molnar 2002). For similar reasons, women face a penalty when navigating the workplace because of motherhood, when other workers suspect they might benefit from favouritism (Duguid et al. 2012) or perceived family‐work conflicts for their competing roles in the household and at work (Nohe et al. 2014). These conflicts grow against the backdrop of Chinese patriarchal normative expectations of women to commit themselves to domestic labour at the *exclusion* of paid labour (Wolf 1984).

Private firms, whether privately held or publicly listed on stock exchanges, remain majority owned by private shareholders who fulfil most of their financing needs. Where state firms are characterized by a firm logic that prioritizes the needs of the public and their employees, private firms are pushed to prioritize the fiduciary interests of their private shareholders. Fligstein and Goldstein (2022) conceptualize this private firm logic as an obsession with short‐term profitability and financial engineering (such as accounting practices to mask losses in a quarter) in order to bolster the company's share price (see also Jung and Dobbin 2015). Moreover, private firms are typically isomorphic in order to be competitive, such that firm logics and employee management practices often spread across entire markets (Gathmann et al. 2022).

Although private firms are more susceptible to layoffs than state firms, Becker (1957) seminally argued that private firms are less discriminatory (toward women and minorities) in order to survive, because discrimination yields higher unit net costs. Gender discrimination amounts to incorporating qualities that are not relevant to productivity. For private firms whose survival depends on productivity, weeding out employees based on their gender alone would detract from running firm operations efficiently and predispose firms to failure (Pager 2016). Cognizant of these risks, private firms should strive to dismantle patriarchal beliefs in the workplace and seek to employ workers based on merit alone.

Indeed, contemporary women are challenging these social boundaries and traditional notions of femininity (Aksu et al. 2025). Women are not passive recipients of patriarchal norms, even if their resistance may often be minimized in the context of the workplace, where boundary distinctions are institutionalized, and men hold superior decision‐making authority over their careers. In particular, many women challenge prevailing societal expectations of men and express a desire for alternative forms of masculinity that emphasize emotional openness and shared responsibilities. These findings highlight that women workers, especially those in private firms, may reject traditional expectations not only for strategic reasons, but because they hold competing visions of gender relations shaped by workplace inequality (Wu and Dong 2019).

This feminist turn carries implications for private firms said to be internationalized into gender‐egalitarian norms, giving social space to women to challenge symbolic boundaries and disrupt the gender stereotypicality of job tasks within them. The rise of women entrepreneurs in the private Internet sector is evidence of this disruption, as women pursue independent careers outside of the household (Cong et al. 2024). Indeed, women may be more active agents in reshaping cultural understandings of gender roles, often in direct response to the prevailing discriminatory environments in Chinese organizations writ large.

As a result, this article hypothesizes that:

Hypothesis 2 In private firms, women are associated with a lower likelihood of possessing the belief that women should be stay‐at‐home wives instead of working.

## 3 Data and Methods

This article uses the Chinese General Social Survey (CGSS), a nationally representative dataset administered by the Chinese Statistics Bureau that targets civilian Chinese adults aged 18 or older. The present study uses pooled data from two waves: the 2017 wave (*N* = 12,528) and the 2021 wave (*N* = 8,148). Representative of the general population in China, the CGSS regularly surveys more than 10,000 households in China on a battery of questions about social structures, social networks, attitudes about inequality, and experiences related to quality of life. Questionnaires are administered face‐to‐face for about 90 min, that ask about attitudes toward women and colleagues. The sampling framework followed a multi‐stage stratified design, covering all regional, geo‐administrative, and county‐level strata (Bian and Li 2012). Survey participants were aged 18 or older.

To test for preferences for women to be stay‐at‐home wives compared to working as the outcome variable, this study draws on the CGSS question, “please indicate the extent to which you agree with this statement: it is better that women get married instead of working.” Responses were ordered on a 5‐point Likert scale: (1) strongly disagree, (2) disagree, (3) neither agree nor disagree, (4) agree, and (5) strongly agree. A higher score indicates a stronger belief that women should marry and be relegated to the household instead of working.

*Socioeconomic resources*.—Socioeconomic resources were conceptualized as independent variables based on (a) income, (b) education, (c) job security, and (d) job autonomy. Based on the total income a respondent received over the past twelve months, they were classified into the following income groups: (1) low class (≤ RMB25,000 per year), (2) lower middle class (RMB25,001–40,000 per year), (3) upper middle class (RMB40,001–100,000 per year), (4) upper class (RMB100,001–200,000 per year), (5) and elite (>RMB200,000 per year).

The highest level of formal education for individuals was also recorded and coded into ordinal classifications: (0) no education, (1) high school or below, (2) technical college, (3) university, (4) postgraduate or above.

Capturing the job security that respondents had at the time of the survey, this study included the CGSS question, “What is your current employment type (full‐time, part‐time, etc.)?” and coded responses into (1) part‐time (or not full‐time) and (2) full‐time.

Similarly, this study accounted for job autonomy using the CGSS question, “for your current job, what role do you take on in terms of management?” Responses were recoded into ordinal classifications: (1) low job autonomy (only managed by others), (2) middle job autonomy (both managed by others and responsible for managing others), and (3) high job autonomy (only responsible for managing others).

*Individual‐level demographic variables*.— Given the prevalence of patriarchal beliefs in China, this article additionally accounts for individual‐level demographic variables that could have self‐selective effects on beliefs about women, namely, *hukou*, *age* and *marital status*.

*Hukou* was controlled for as a categorical variable consisting of four types of registration: urban *hukou*, resident *hukou* but who were previously urban, resident *hukou* but who were previously rural, and rural *hukou*. Nationwide, Chinese citizens are assigned types of citizenship based on their city or locality of birth. Introduced in the 1950s to restrict migration, the most common forms of *hukou* are the urban (*feinongye*) and rural (*nongye*) registrations. In 2014, an experimental policy reform was introduced in select cities, a new integrated “resident” (*jumin*) *hukou* registration, into which citizens are transitioned. Each type of *hukou* determines one's access to welfare, school registration, and even eligibility for job categories, producing restrictions on life chances. As such, *hukou* registrations constitute a unique form of socioeconomic resource, because of their unique effects on life chances. A rich body of evidence identifies a gap in income, occupational mobility, and wellbeing between urban and rural *hukou* (Chen et al. 2018; Song and Smith 2021; Zhong et al. 2022). The relative deficiency of socioeconomic opportunities in rural regions thus amplifies their patriarchal values toward women, constraining educational and work opportunities, compared to urban regions with greater diversity (Au 2024a,b).

Age was recoded into ordinal classifications: (1) aged 18 to 30, (2) aged 31 to 40, (3) aged 41 to 50, (4) aged 51 to 60, (5) aged 61 to 70, (6) aged 71 to 80, (7) aged 81 and above. This design takes stock of evidence that older adults are more inclined to believe in traditional patriarchal values, given that they spent their early lives in a socioeconomically deprived environment (a less prosperous China) with lower educational achievement that is not conducive to postmaterialist and liberalist values (Hu and Scott 2016).

Marital status was additionally recorded as (1) married or (0) not married. Married individuals may be more likely to express gendered traditional beliefs in favour of marriage, because of self‐selective effects. Having elected to get married themselves, they likely hold a more favourable view of marriage reflective of the choice they themselves have made, compared to those who have not married.

*Firm‐level effects*, namely, firm size, were also controlled for. Just like firm type, the size of a firm is correlated with organizational cultures more or less tolerant toward women. The evidence, however, is mixed on this score. Some studies have identified larger firms as less discriminatory, because larger firms are likely to be under watch by a greater number of stakeholders. Stakeholder oversight, in turn, leads to the explicit enforcement of anti‐discriminatory policies and pressure to improve female representation per corporate social governance principles (Knight et al. 2022; Patel & Yates 2023). Others, however, have argued that larger firms are led by male‐dominated boards who are unwilling to hire women, done with the implicit backing of large shareholders (Post and Byron 2015). Specific evidence from China, for instance, finds that large firms are hotbeds of discrimination against women's employment (Gao et al. 2016). Firm size was thus controlled for and classified into: (1) micro firms (fewer than 10 employees), (2) small firms (10 to 49 employees), (3) medium firms (50 to 249 employees), (4) large firms (250 to 9,999 employees), and (5) mega firms (10,000 employees or more).

The sample was stratified into two subsamples based on the type of firm in which respondents were employed at the time of the survey. The analytic subsample includes only employed respondents working in organizations classified by CGSS as state‐owned or private firms. Individuals who were unemployed, self‐employed, agricultural workers, or outside the labour force were excluded. The final analytic sample includes 1,897 state‐firm employees, including SOEs and COEs, and 2,488 private‐firm employees. This enabled a fine‐grained examination of how predictors of belief about women's employment varied across genders and firm types. Ordinal logistic regressions were conducted on beliefs about women's employment. I regress patriarchal beliefs about women *Y_gb_* against socioeconomic resources *Y_inc_, Y_edu_, Y_jsec_, Y_jau_*, *hukou* registration *Y_huk_*, and controlling for *Y_age_*, *Y_mar_*, firm size *Y_firm_*, and *Y_year_*. This is expressed as:

(1) $$\begin{array}{ccc} \;Y_{gb} & = & \alpha +\beta_{1}Y_{inc}+\beta_{2}Y_{edu}+\beta_{3}Y_{jsec}+\beta_{4}Y_{jau}+\beta_{5}Y_{huk}\\ & & +\;\beta_{6}Y_{age}+\beta_{7}Y_{mar}+\beta_{8}Y_{firm}+\beta_{9}Y_{year}+\varepsilon \end{array}$$

Logistic regressions on ordinal conceptualizations create parameter estimates with low bias and high efficiency, allowing a straightforward interpretation of the odds of belief (support) that women should marry instead of work (Riedl and Geishecker 2014). Missing values were handled using listwise deletion.

In addition, I statistically compare differences in belief between state and private firm employees using unequal variance *t*‐tests (Ruxton 2006), given by:

(2) $$t=\frac{\mu_{1}-\mu_{2}}{\sqrt{\frac{s_{1}^{2}}{n_{1}}+\frac{s_{2}^{2}}{n_{2}}}}\;$$

where μ_j_ is the mean of group *j*, s*_j_* indicates the variance, and n*_j_* is the size of group *j*. Unequal variance *t*‐tests are ideal for groups of different sizes, as with groups of state and private firm employees.

## 4 Results

Table 1 presents the descriptive statistics for the whole sample. Employees in state and private firms are generally comparable in terms of age groups, proportion of women workers, *hukou* registrations, job security, marital statuses, degree of job autonomy, and work in comparably sized firms (Table 1).

**TABLE 1:** Mean (standard deviation) of covariates by firm type.

	State firms	Private firms
Gender		
Men	959	938
	(43.0%)	(43.53%)
Women	1,271	1,217
	(57.0%)	(56.47%)
Age group		
18–30	346	646
	(18.2%)	(26.0%)
31–40	398	556
	(21.0%)	(22.3%)
41–50	430	496
	(22.7%)	(19.9%)
51–60	382	402
	(20.1%)	(16.2%)
61–70	217	263
	(11.4%)	(10.6%)
71–80	100	103
	(5.3%)	(4.1%)
81+	24	22
	(1.3%)	(0.9%)
Mean age group	2.79	3.06
	(1.51)	(1.52)
Highest education		
No education	118	131
	(6.2%)	(5.3%)
High school or below	416	898
	(21.9%)	(36.1%)
Technical college	560	710
	(29.5%)	(28.5%)
University	715	705
	(37.7%)	(28.3%)
Postgraduate or above	88	44
	(4.6%)	(1.8%)
Mean education	2.13	1.85
	(1.01)	(0.951)
Income group		
Low class	327	616
	(18.0%)	(26.0%)
Lower middle class	338	498
	(18.6%)	(21.0%)
Upper middle class	869	948
	(47.7%)	(40.0%)
Upper class	215	205
	(11.8%)	(8.6%)
Elite	72	105
	(4.0%)	(4.4%)
Mean income group	2.65	2.45
	(1.03)	(1.10)
Marital status	0.810	0.726
	(0.392)	(0.446)
Firm size		
Micro	275	747
	(18.5%)	(37.1%)
Small	466	645
	(31.4%)	(29.5%)
Medium	551	431
	(37.2%)	(32.0%)
Large	179	182
	(12.1%)	(21.4%)
Mega	12	8
	(0.8%)	(0.4%)
Mean firm size	2.42	1.96
	(1.02)	(1.10)
Job security	1.96	1.93
	(0.205)	(0.251)
Job autonomy	1.92	1.86
	(0.797)	(0.834)
*Hukou*		
Rural	448	1,148
	(24.0%)	(46.3%)
Resident (previously rural)	351	275
	(18.8%)	(11.1%)
Resident (previously urban)	364	369
	(19.5%)	(14.9%)
Urban	704	689
	(37.7%)	(27.8%)
*N*	1,897	2,488

There are minor differences, whereby private firm employees appear to be older in age on average than state firm employees, but less educated than the latter. There appear to be more women than men in both types of firms, which invites a comparison of their socioeconomic resources within each firm type.

**FIGURE 1:**
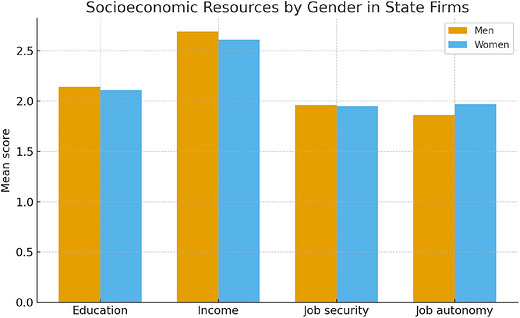
Results of *t*‐tests comparing socioeconomic resources between men and women among state firms. [Colour figure can be viewed at wileyonlinelibrary.com]

**FIGURE 2:**
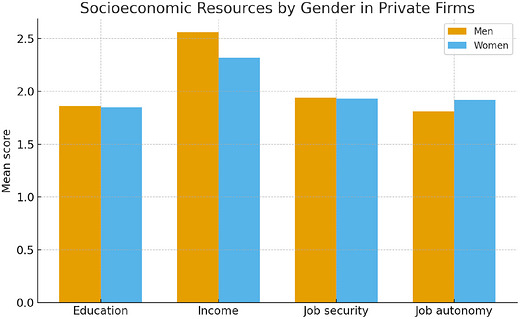
Results of *t*‐tests comparing socioeconomic resources between men and women among private firms. [Colour figure can be viewed at wileyonlinelibrary.com]

Using Equation (2), Figures 1 and 2 present the *t*‐test results comparing the distribution of socioeconomic resources among men and women within state and private firms. In state firms (Figure 1), we observe that women (*x̄* = 1.97) have statistically significant greater job autonomy than their male counterparts (*x̄* = 1.86), contrary to observations that women suffer pay disparities compared to their male counterparts in China (Wang and Cheng 2021; Wu and Qi 2017).

In private firms (Figure 2), we observe that women (*x̄* = 1.92) also have significantly greater job autonomy than men (*x̄* = 1.81). However, men (*x̄* = 1.86) also have a statistically significant lead in their educational achievement than women (*x̄* = 1.85), though the difference is comparatively small.

Using Equation (1), the statistical analyses add nuance to examining differences in the effect of socioeconomic resources on the belief that women should be stay‐at‐home wives instead of working across state and private firms. Table 2 first parses out the effects of different predictors across the entire sample. Private firm employment is indeed associated with significantly higher odds (OR = 1.302) of supporting the belief that women should be stay‐at‐home wives instead of working. Being female is associated with lower odds of supporting the belief that women should be stay‐at‐home wives instead of working (OR = 0.753). However, socioeconomic resources generally diminish the odds of this belief, supporting Hypothesis 1: income is associated with lower odds (OR = 0.819) of supporting this belief, as is resident *hukou* compared to rural *hukou* (OR = 0.816).

**TABLE 2:** Regressing belief that women should marry and stay at home instead of work among all firms.

Predictor	Odds Ratio (Standard Error)
Private firm	1.302^***^ (0.069)
Female	0.753^***^ (0.065)
Highest education	0.949 (0.038)
Age group	0.986 (0.025)
Income group	0.819^***^ (0.033)
*Hukou* (ref: rural)	
Resident (previously rural)	0.816^*^ (0.102)
Resident (previously urban)	1.040 (0.101)
Urban	0.928 (0.082)
Married	1.508^***^ (0.080)
Job security	1.101 (0.155)
Job autonomy	0.920 (0.045)
Firm size	0.996 (0.033)

^*^ *P* < .05; ^**^ *p* < .01; ^***^ *p* < .001

In state firms (Table 3), a different set of socioeconomic resources appears to matter: income is once more associated with lower odds of the belief that women should be stay‐at‐home wives instead of working (OR = 0.800), whereas education and *hukou* registrations are not significant, partially supporting Hypothesis 1. However, having more job autonomy emerges as a predictor of lower support for the belief that women should be stay‐at‐home wives instead of working (OR = 0.826). Women themselves, moreover, do not appear to have any significant association with this belief.

**TABLE 3:** Regressing belief that women should marry and stay at home instead of work by firm type.

	State firms	Private firms
Predictor	Odds Ratio (Standard Error)	Odds Ratio (Standard Error)
Female	0.856 (0.099)	0.685^***^ (0.052)
Highest education	0.906 (0.056)	0.976 (0.034)
Age group	0.946 (0.039)	1.015 (0.335)
Income group	0.800^***^ (0.054)	0.817^***^ (0.043)
*Hukou* (ref: rural)		
Resident (previously rural)	0.776 (0.161)	0.959 (0.140)
Resident (previously urban)	1.157 (0.163)	0.989 (0.131)
Urban	0.988 (0.137)	0.891 (0.105)
Married	1.513^**^ (0.136)	1.488^***^ (0.099)
Job security	0.816 (0.306)	1.239 (0.179)
Job autonomy	0.826^**^ (0.070)	1.001 (0.594)
Firm size	1.066 (0.052)	0.941 (0.042)

^*^ *P* < .05; ^**^ *p* < .01; ^***^ *p* < .001

In private firms, we observe that being female is associated with lower odds of the belief that women should be stay‐at‐home wives instead of working (OR = 0.685). Income is once more associated with significantly lower odds of possessing this belief against women's employment among employees (OR = 0.817). However, there is no influence from education, *hukou* registrations, or job resources on the odds of the belief that women should be stay‐at‐home wives instead of working.

Because coefficients from nonlinear models are not directly comparable across samples (Breen et al. 2018), we refrain from interpreting cross‐model differences in coefficient magnitudes. Instead, we focus on the direction, statistical significance, and predicted patterns within each firm type. It appears that in private firms, there are fewer socioeconomic resource variables that are capable of significantly lowering patriarchal beliefs against women in the workplace.

**FIGURE 3:**
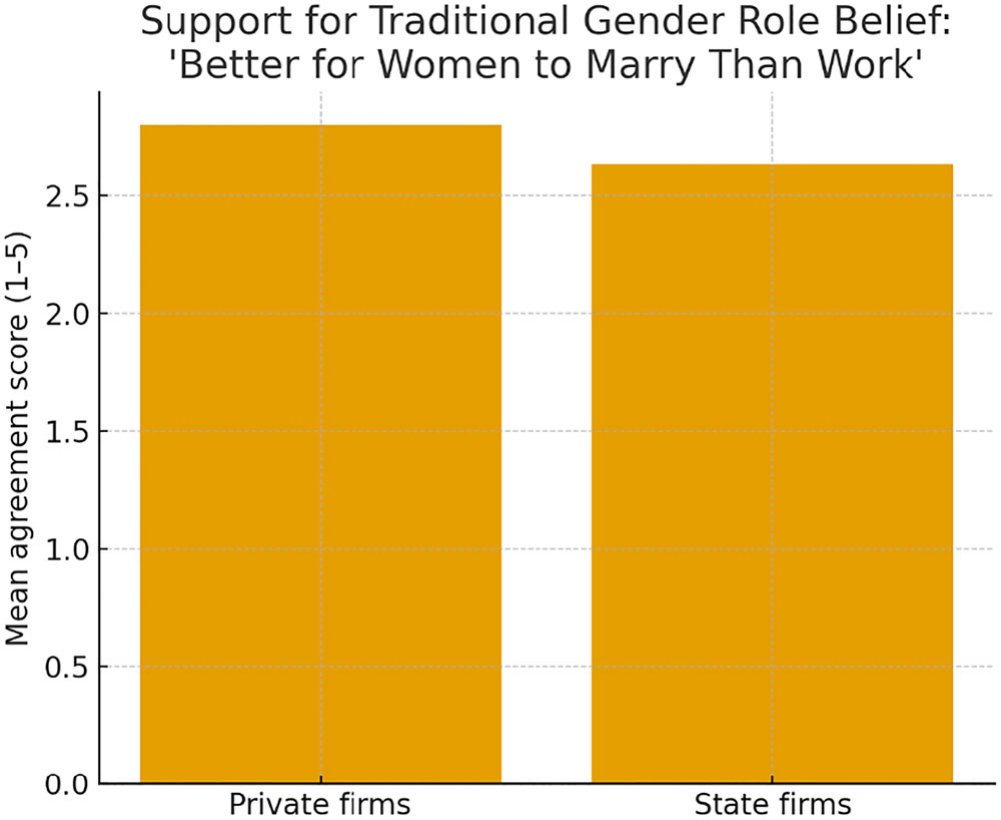
Results of *t*‐tests comparing belief that women should marry instead of work between private and state firms. A higher score indicates a stronger belief that women should marry instead of work. [Colour figure can be viewed at wileyonlinelibrary.com]

Figure 3 complements these results on socioeconomic resources by directly examining the belief that women should be stay‐at‐home wives instead of working within these firm types. The results reveal statistically significant differences between employees of the two firm types: private firm employees appear to agree with the belief that women should be stay‐at‐home wives instead of working (*x̄* = 2.80) to a much greater extent than their state firm counterparts (*x̄* = 2.63), which does not support Hypothesis 2.

## 5 Discussion

This article provides an important sociological account of contemporary gender disparities in Chinese firms. Contrary to research that suggests women are materially deprived in socioeconomic resources compared to men, this study observes few statistically significant differences in income, education, job security, and job autonomy between men and women. In fact, the results suggest that women have an even higher degree of job autonomy than men in both state and private firms.

This article identifies that heterogeneous types of socioeconomic resources wield differential effects on the belief that women should be stay‐at‐home wives instead of working between the two firm types, offering support for Hypothesis 1. At the aggregate level in all firms, having a resident *hukou* registration predicts lower odds of supporting the belief that women should be stay‐at‐home wives instead of working compared to a rural *hukou*. Simultaneously, income is a powerfully consistent predictor of lower odds of supporting the belief that women should be stay‐at‐home wives instead of working in state and private firms at the subsample level. Job autonomy also predicts lower odds of this belief, but only in state firms.

Contrary to expectations, there are fewer forms of socioeconomic resources that are capable of motivating workers to lessen their patriarchal beliefs against women in the workplace in private firms (only income) compared to state firms (income *and* job autonomy). More directly, the results reveal that private firm workers profess stronger patriarchal beliefs against women's employment than their state firm counterparts. I theorize this may be why women also appear to be more agitated about patriarchal beliefs when they work in private firms, but not in state firms. To illustrate, women are opposed to the belief that women should be stay‐at‐home wives instead of working in private firms, but not in state firms. This suggests that women are not passive recipients of patriarchal norms. Research in comparable patriarchal contexts (Aksu et al. 2025) shows that women articulate emerging ideals of masculinity emphasizing emotional openness and shared responsibility. These findings highlight that women workers, especially those in private firms, may reject traditional expectations not only for strategic reasons, but because they hold competing visions of gender relations shaped by workplace inequality.

The findings suggest that private firms are not the anti‐discriminatory workplaces they are believed to be, but seedbeds for patriarchal beliefs, especially as women appear to achieve greater job autonomy. Why, despite women's gains in employment and nationwide economic development, do workers still believe that women should be stay‐at‐home wives instead of working?

This work gains credence from and contributes to the literature on boundaries and group threat in organizations (Olson et al. 2007). Where much of this work has focused on racial and ethnic relations, the present article extends this dialogue to examine how men may be disgruntled with women getting ahead and subsequently react in punitive ways in Chinese firms. These findings align with research showing that perceived exclusion strengthens adherence to traditional norms (Borinca et al. 2025). In competitive private firms, men may perceive women's rising autonomy as encroaching on historically male‐dominated spaces, activating identity‐based defensive responses. Relatedly, women's stronger rejection of patriarchal norms in private firms is consistent with studies showing women articulate alternative visions of gender relations when confronting unequal treatment (Aksu et al. 2025).

The present study highlights overlooked organizational contexts of gender discrimination where additional oversight is required. Private firms are left to their own devices under the auspices of market‐driven reforms in China, which dissuades extensive government interventions for misconduct. This, however, runs the risk of obscuring the extent of gender discrimination—risks that the present study reveals to be real and pressing.

Just as important, this article finds that women appear to be more empowered to resist patriarchal norms and gender stereotypicality in job roles, evinced by their willingness to express disagreement with the belief that they should simply marry and be stay‐at‐home wives. The evidence suggests an important portrait of women as not passive recipients of patriarchal beliefs, but prospective co‐creators of the symbolic boundaries that isolate them in the workplace (Lamont & Molnar 2002).

Recent discrimination cases uncovered at prominent information technology companies in China are telling of increasing awareness of gendered biases—and willingness to report it. Alibaba, an e‐commerce firm and China's second most valuable company, was found by the Human Rights Watch (2018) to engage in discriminatory hiring practices against women. Exemplar job ads from Alibaba requested female candidates to “possess fine personal image and qualities, [including]… physical characteristics like those of popular female Japanese porn star Sola Aoi” (p.38). In response, thousands of female Chinese tech workers signed petitions to urge the police and the company to investigate the incident (Liu 2023). Given that the Internet sector in China is one of the most prolific sources of employment nationwide, representing close to 30% of economic output, they comprise a significant proxy for understanding female employee treatment within private firms at large.

Finally, although Becker (1957) argues that firms are less discriminatory towards women to have an advantage for survival, this article finds that private firms constitute organizational contexts that warrant additional monitoring, such as expanding meeting channels to review and evaluate compliance measures between firm directors and the Ministry of Human Resources and Social Security. The results suggest the importance of monitoring mechanisms, such as periodic audits of recruitment advertisements and third‐party reporting channels, particularly in private firms.

## 6 Limitations and Future Directions

Although this study advances our understanding of patriarchal beliefs in Chinese firms, several limitations should be noted. First, the cross‐sectional nature of the data precludes causal inferences about how socioeconomic resources or firm ownership types may shape beliefs over time. The results thus offer a starting point for future longitudinal analyses, which could track belief changes amid economic shifts. This is especially important, though challenging, given the paucity of longitudinal data sufficient to track changes throughout the life course in advanced societies (Guinea‐Martin et al. 2018).

Second, China is largely a racially homogeneous society that is characteristic of East Asian societies. While the results offer a useful point of comparison for other East Asian societies, future studies outside of East Asia have room to feature intersectional analyses that incorporate ethnicity or class alongside gender, which would enrich our understanding of how inequality compounds.

Third, the sample focuses on employed individuals in state and private firms, limiting generalizability to informal sectors or unemployed populations, where gender norms may differ. Finally, the reliance on quantitative measures captures broad patterns but overlooks nuanced interactions, such as how beliefs translate into discriminatory behaviours in daily organizational life. Qualitative studies, such as ethnographic observations or in‐depth interviews in firms, would illuminate micro‐level processes of symbolic boundary‐policing and discrimination.

## Funding

This research was funded by a Departmental Start‐up Grant (P0040982) and Applied Social Sciences Research Grant (P0046151) from the Department of Applied Social Sciences at The Hong Kong Polytechnic University.

## References

[cars70043-bib-0001] Aksu, A. , Y. Koc , I. Borinca , and S. Otten . 2025. “Beyond Traditional Masculinities: Women's Perceptions of new Masculinities.” Psychology of Men & Masculinities 26, no. 3: 384–399.

[cars70043-bib-0002] Alegria, S. 2019. “Escalator or Step Stool? Gendered Labor and Token Processes in Tech Work.” Gender & Society 33, no. 5: 722–745.

[cars70043-bib-0003] Alegria, S. , and E. Branch . 2015. “Causes and Consequences of Inequality in the STEM: Diversity and its Discontents.” International Journal of Gender, Science and Technology 7, no. 3: 321–342.

[cars70043-bib-0004] Alfrey, L. , and F. Twine . 2017. “Gender‐Fluid Geek Girls: Negotiating Inequality Regimes in the Tech Industry.” Gender & Society 31, no. 1: 28–50.

[cars70043-bib-0005] Au, A. 2024a. “Attitudes Toward Women's Layoffs During Recessions: Evidence From Chinese Firms.” Socius: Sociological Research for a Dynamic World 10: 23780231241266733.

[cars70043-bib-0006] Au, A. 2024b. “Digitalization in China: Who's Left behind?.” Information, Communication & Society 27, no. 6: 1247–1265.

[cars70043-bib-0007] Bansak, K. , J. Hainmueller , and D. Hangartner . 2016. “How Economic, Humanitarian, and Religious Concerns Shape European Attitudes Toward Asylum Seekers.” Science 354, no. 6309: 217–222. 27708060 10.1126/science.aag2147

[cars70043-bib-0008] Becker, G. 1957. The Economics of Discrimination. University of Chicago Press.

[cars70043-bib-0009] Bian, Y. , and L. Li . 2012. “The Chinese General Social Survey (2003–8) Sample Designs and Data Evaluation.” Chinese Sociological Review 45, no. 1: 70–97.

[cars70043-bib-0010] Biemann, T. , H. Zacher , and D. C. Feldman . 2012. “Career Patterns: A Twenty‐Year Panel Study.” Journal of Vocational Behavior 81, no. 2: 159–170.

[cars70043-bib-0011] Borinca, I. , R. Guerra , and F. Uka . 2025. ““Ins and Outs”: Ethnic Identity, the Need to Belong, and Responses to Inclusion and Exclusion in Inclusive Common Ingroups.” Group Processes & Intergroup Relations 28, no. 2: 324–354.

[cars70043-bib-0012] Bourdieu, P. 1984. Distinction: A Social Critique of the Judgment of Taste. Routledge.

[cars70043-bib-0013] Breen, R. , K. B. Karlson , and A. Holm . 2018. “Interpreting and Understanding Logits, Probits, and Other Nonlinear Probability Models.” Annual Review of Sociology 44, no. 1: 39–54.

[cars70043-bib-0015] Burt, R. 2019. “The Networks and Success of Female Entrepreneurs in China.” Social Networks 58: 37–49.

[cars70043-bib-0016] Campero, S. 2021. “Hiring and Intra‐Occupational Gender Segregation in Software Engineering.” American Sociological Review 86, no. 1: 60–92.

[cars70043-bib-0017] Census and Statistics Department of Hong Kong . 2023. Women and Men in Hong Kong—Key Statistics: 2023 Edition. Census and Statistics Department of Hong Kong.

[cars70043-bib-0018] Charles, M. , R. Friedland , J. Afary , and R. Yang . 2023. “Complicating Patriarchy: Gender Beliefs of Muslim Facebook Users in the Middle East North Africa, and South Asia.” Gender & Society 37, no. 1: 91–123.

[cars70043-bib-0019] Chen, C. , R. LeGates , M. Zhao , and C. Fang . 2018. “The Changing Rural‐Urban Divide in China's Megacities.” Cities 81: 81–90.

[cars70043-bib-0021] Cong, L. , J. Ponticelli , X. Yang , and X. Zhang . 2024. Bridging the Gender Gap in Entrepreneurship and Empowering Women via Digital Technologies . Working Paper. National Bureau of Economic Research.

[cars70043-bib-0022] Correll, S. , S. Benard , and I. Paik . 2007. “Getting a Job: Is There a Motherhood Penalty?.” American Journal of sociology 112, no. 5: 1297–1338.

[cars70043-bib-0023] Correll, S. , K. Weisshaar , A. Wynn , and J. Wehner . 2020. “Inside the Black box of Organizational Life: The Gendered Language of Performance Assessment.” American Sociological Review 85, no. 6: 1022–1050.

[cars70043-bib-0024] De Coninck, D. 2020. “Migrant Categorizations and European Public Opinion: Diverging Attitudes Towards Immigrants and Refugees.” Journal of Ethnic and Migration Studies 46, no. 9: 1667–1686.

[cars70043-bib-0025] DiTomaso, N. , and Y. Bian . 2018. “The Structure of Labor Markets in the US and China: Social capital and Guanxi.” Management and Organization Review 14, no. 1: 5–36.

[cars70043-bib-0026] Dobbin, F. , and A. Kalev . 2021. “The Civil Rights Revolution at Work: What Went Wrong.” Annual Review of Sociology 47: 281–303.

[cars70043-bib-0027] Duguid, M. M. , D. L. Loyd , and P. S. Tolbert . 2012. “The Impact of Categorical Status, Numeric Representation, and Work Group Prestige on Preference for Demographically Similar Others: A Value Threat Approach.” Organization Science 23, no. 2: 386–401.

[cars70043-bib-0028] Elliott, S. , and M. Reid . 2019. “Low‐Income Black Mothers Parenting Adolescents in the Mass Incarceration Era: The Long Reach of Criminalization.” American Sociological Review 84, no. 2: 197–219.

[cars70043-bib-0029] Emerson, K. T. , and M. C. Murphy . 2015. “A Company I Can Trust? Organizational Lay Theories Moderate Stereotype Threat for Women.” Personality and Social Psychology Bulletin 41, no. 2: 295–307. 25534242 10.1177/0146167214564969

[cars70043-bib-0030] England, P. , A. Levine , and E. Mishel . 2020. “Progress Toward Gender Equality in the United States has Slowed or Stalled.” Proceedings of the National Academy of Sciences 117, no. 13: 6990–6997. 10.1073/pnas.1918891117 PMC7132302 32229559

[cars70043-bib-0031] Epstein, C. F. 2007. “Great Divides: the Cultural, Cognitive, and Social Bases of the Global Subordination of Women.” American Sociological Review 72, no. 1: 1–22.

[cars70043-bib-0032] Ferree, M. 2020. “The Crisis of Masculinity for Gendered Democracies: Before, During, and After Trump.” Sociological Forum 35: 898–917.

[cars70043-bib-0033] Fligstein, N. , and A. Goldstein . 2022. “The Legacy of Shareholder Value Capitalism.” Annual Review of Sociology 48: 193–211.

[cars70043-bib-0098] Gathmann, C. , I. Helm , and U. Schönberg . 2020. “Spillover effects of mass layoffs.” Journal of the European Economic Association 18, no. 1: 427–468.

[cars70043-bib-0035] Gao, H. , Y. Lin , and Y. Ma . 2016. “Sex Discrimination and Female Top Managers: Evidence From China.” Journal of Business Ethics 138: 683–702.

[cars70043-bib-0037] Gonalons‐Pons, P. , and M. Gangl . 2021. “Marriage and Masculinity: Male‐Breadwinner Culture, Unemployment, and Separation Risk in 29 Countries.” American Sociological Review 86, no. 3: 465–502. 34149053 10.1177/00031224211012442 PMC8211126

[cars70043-bib-0038] Guinea‐Martin, D. , R. Mora , and J. Ruiz‐Castillo . 2018. “The Evolution of Gender Segregation Over the Life Course.” American Sociological Review 83, no. 5: 983–1019.

[cars70043-bib-0039] Guo, Y. , Q. Huy , and Z. Xiao . 2017. “How Middle Managers Manage the Political Environment to Achieve Market Goals: Insights From China's State‐Owned Enterprises.” Strategic Management Journal 38, no. 3: 676–696.

[cars70043-bib-0040] Gorman, E. H. 2005. “Gender Stereotypes, Same‐Gender Preferences, and Organizational Variation in the Hiring of Women: Evidence From Law Firms.” American Sociological Review 70, no. 4: 702–728.

[cars70043-bib-0041] Heilman, M. , F. Manzi , and S. Caleo . 2019. “Updating Impressions: the Differential Effects of New Performance Information on Evaluations of Women and Men.” Organizational Behavior and Human Decision Processes 152: 105–121.

[cars70043-bib-0042] Hu, Y. , and J. Scott . 2016. “Family and Gender Values in China: Generational, Geographic, and Gender Differences.” Journal of Family Issues 37, no. 9: 1267–1293.

[cars70043-bib-0043] Human Rights Watch . 2018. “Only Men Need Apply” Gender Discrimination in Job Advertisements in China. *Human Rights Watch* . https://www.hrw.org/report/2018/04/23/only-men-need-apply/gender-discrimination-job-advertisements-china

[cars70043-bib-0044] Jones, L. , R. Manuelli , and E. McGrattan . 2015. “Why are Married Women Working so Much?.” Journal of Demographic Economics 81, no. 1: 75–114.

[cars70043-bib-0045] Jung, J. , and F. Dobbin . 2015. “Agency Theory as Prophecy: How Boards, Analysts, and Fund Managers Perform Their Roles.” Seattle University Law Review 39: 291–320.

[cars70043-bib-0046] Kaufman, J. 2004. “Endogenous Explanation in the Sociology of Culture.” Annual Review of Sociology 30: 355–387.

[cars70043-bib-0047] Kmec, J. A. , S. McDonald , and L. Trimble . 2010. “Making Gender fit and “Correcting” Gender Misfits: Sex Segregated Employment and the Nonsearch Process.” Gender & Society 24, no. 2: 213–236.

[cars70043-bib-0048] Knight, C. , F. Dobbin , and A. Kalev . 2022. “Under the Radar: Visibility and the Effects of Discrimination Lawsuits in Small and Large Firms.” American Sociological Review 87, no. 2: 175–201.

[cars70043-bib-0049] Lamont M. and Fournier M. (Eds.). 1992. Cultivating Differences: Symbolic Boundaries and the Making of Inequality. University of Chicago Press.

[cars70043-bib-0050] Lamont, M. , and V. Molnár . 2002. “The Study of Boundaries in the Social Sciences.” Annual Review of Sociology 28, no. 1: 167–195.

[cars70043-bib-0051] Lee, M. , and L. Huang . 2018. “Gender Bias, Social Impact Framing, and Evaluation of Entrepreneurial Ventures.” Organization Science 29, no. 1: 1–16.

[cars70043-bib-0097] Lin, N. 2001. Social Capital: A Theory of Social Structure and Action. Cambridge: Cambridge University Press.

[cars70043-bib-0053] Liu, H. Y. 2023. “When Nobody Listens, go Onlineʼʼ: The “807” Labor Movement Against Workplace Sexism in China's Tech Industry.” Gender, Work & Organization 30, no. 1: 312–328.

[cars70043-bib-0054] Liu, J. 2004. “Holding up the Sky? Reflections on Marriage in Contemporary China.” Feminism & Psychology 14, no. 1: 195–202.

[cars70043-bib-0055] Lizardo, O. , and M. Strand . 2010. “Skills, Toolkits, Contexts and Institutions: Clarifying the Relationship Between Different Approaches to Cognition in Cultural Sociology.” Poetics 38, no. 2: 205–228.

[cars70043-bib-0056] Ludwig, V. , and J. Brüderl . 2018. “Is There a Male Marital Wage Premium?ʼʼ New Evidence From the United States.” American Sociological Review 83, no. 4: 744–770.

[cars70043-bib-0057] Manzi, F. , and M. Heilman . 2021. “Breaking the Glass Ceiling: for One and All?.” Journal of Personality and Social Psychology 120, no. 2: 257–277. 33252976 10.1037/pspa0000260

[cars70043-bib-0058] Mao, T. 1953. Report of an Investigation Into the Peasant Movement in Hunan. Foreign Languages Press.

[cars70043-bib-0059] National Bureau of Statistics of China . 2022. 2022 China Statistical Yearbook. National Bureau of Statistics of China.

[cars70043-bib-0060] Nohe, C. , A. Michel , and K. Sonntag . 2014. “Family–Work Conflict and Job Performance: A Diary Study of Boundary Conditions and Mechanisms.” Journal of Organizational Behavior 35, no. 3: 339–357.

[cars70043-bib-0061] Olson, J. , I. Frieze , S. Wall , et al. 2007. “Beliefs in Equality for Women and Men as Related to Economic Factors in Central and Eastern Europe and the United States.” Sex Roles 56: 297–308.

[cars70043-bib-0062] Organisation for Economic Co‐operation and Development (OECD) . 2022. Social Institutions & Gender Index . Organisation for Economic Co‐operation and Development.

[cars70043-bib-0063] Organisation for Economic Co‐operation and Development (OECD) . 2023. OECD Economic Outlook, Interim Report , *March 2023*. Organisation for Economic Co‐operation and Development.

[cars70043-bib-0064] Pager, D. 2016. “Are Firms That Discriminate More Likely to Go out of Business?.” Sociological Science 3: 849–859.

[cars70043-bib-0065] Palmer, M. 1995. “The Re‐Emergence of Family Law in Post‐Mao China: Marriage, Divorce and Reproduction.” The China Quarterly 141: 110–134.

[cars70043-bib-0066] Patel, D. , and S. Yates . 2023. “How Does Firm Size and Sector Impact Female and Minority Representation.” The Journal of Business Diversity 23, no. 2: 52–63.

[cars70043-bib-0067] Pepin, J. , and D. Cotter . 2018. “Separating Spheres? Diverging Trends in Youth's Gender Attitudes About Work and family.” Journal of Marriage and Family 80, no. 1: 7–24.

[cars70043-bib-0068] Post, C. , and K. Byron . 2015. “Women on Boards and Firm Financial Performance: A Meta‐Analysis.” Academy of Management Journal 58, no. 5: 1546–1571.

[cars70043-bib-0069] Quadlin, N. , and L. Doan . 2018. “Sex‐Typed Chores and the City: Gender, Urbanicity, and Housework.” Gender & Society 32, no. 6: 789–813.

[cars70043-bib-0070] Quillian, L. 1996. “Group Threat and Regional Change in Attitudes Toward African‐Americans.” American Journal of sociology 102, no. 3: 816–860.

[cars70043-bib-0071] Reskin, B. , and P. A. Roos . 1990. Job Queues, Gender Queues: Explaining Women's Inroads Into Male Occupations. Temple University Press.

[cars70043-bib-0072] Riedl, M. , and I. Geishecker . 2014. “Keep It Simple: Estimation Strategies for Ordered Response Models With Fixed Effects.” Journal of Applied Statistics 41, no. 11: 2358–2374.

[cars70043-bib-0073] Risman, B. 2004. “Gender as a Social Structure: Theory Wrestling With Activism.” Gender & Society 18, no. 4: 429–450.

[cars70043-bib-0074] Rivera, L. , and A. Tilcsik . 2019. “Scaling Down Inequality: Rating Scales, Gender Bias, and the Architecture of Evaluation.” American Sociological Review 84, no. 2: 248–274.

[cars70043-bib-0075] Rosenstein, J. E. 2008. “Individual Threat, Group Threat, and Racial Policy: Exploring the Relationship Between Threat and Racial Attitudes.” Social Science Research 37, no. 4: 1130–1146. 19227695 10.1016/j.ssresearch.2008.04.001

[cars70043-bib-0076] Ruxton, G. D. 2006. “The Unequal Variance T‐Test Is an Underused Alternative to Student's T‐Test and the Mann–Whitney U Test.” Behavioral Ecology 17, no. 4: 688–690.

[cars70043-bib-0077] Seeberg, V. , and S. Luo . 2018. “Migrating to the City in Northwest China: Young Rural Women's Empowerment.” Journal of Human Development and Capabilities 19, no. 3: 289–307.

[cars70043-bib-0078] Shen, J. , and Q. Wang . 2023. “Do Men and Women Discriminate Against Women for the Same Reason?ʼʼ Evidence From China.” China Economic Review 77: 101908.

[cars70043-bib-0079] Smith‐Doerr, L. , S. Alegria , K. Husbands Fealing , D. Fitzpatrick , and D. Tomaskovic‐Devey . 2019. “Gender Pay Gaps in US Federal Science Agencies: An Organizational Approach.” American Journal of sociology 125, no. 2: 534–576.

[cars70043-bib-0081] Song, Q. , and J. Smith . 2021. “The Citizenship Advantage in Psychological Well‐Being: An Examination of the Hukou System in China.” Demography 58, no. 1: 165–189. 33834239 10.1215/00703370-8913024 PMC10186556

[cars70043-bib-0082] Stein, A. 2001. The Stranger Next Door: The Story of a Small Community's Battle over Sex, Faith, and Civil Rights.Beacon Press.

[cars70043-bib-0200] Swidler, A. 2003. Talk of love: How culture matters. Chicago, IL: University of Chicago Press.

[cars70043-bib-0083] Vaisey, S. 2008. “Socrates, Skinner, and Aristotle: ‘Three Ways of Thinking About Culture in Action’.” Sociological Forum 23, no. 3: 603–613.

[cars70043-bib-0084] Vaisey, S. , and A. Miles . 2014. “Tools From Moral Psychology for Measuring Personal Moral Culture.” Theory and Society 43, no. 3: 311–332.

[cars70043-bib-0085] Von Hippel, C. , M. Issa , R. Ma , and A. Stokes . 2011. “Stereotype Threat: Antecedents and Consequences for Working Women.” European Journal of Social Psychology 41, no. 2: 151–161.

[cars70043-bib-0086] Wang, D. , and X. Luo . 2019. “Retire in Peace: Officials' Political Incentives and Corporate Diversification in China.” Administrative Science Quarterly 64, no. 4: 773–809.

[cars70043-bib-0087] Wang, H. , and Z. Cheng . 2021. “Mama Loves You: The Gender Wage Gap and Expenditure on Children's Education in China.” Journal of Economic Behavior & Organization 188: 1015–1034.

[cars70043-bib-0088] Wang, O. 2022. China's state Firms Needed to Provide ‘Economic Foundation’, but Pressure Mounting Due to ‘Hidden losses.’. *South China Morning Post*. Retrieved online: https://www.scmp.com/economy/china‐economy/article/3198859/chinas‐state‐firms‐needed‐provide‐economic‐foundation‐pressure‐mounting‐due‐hidden‐losses.

[cars70043-bib-0089] Wolf, M. 1984. “Marriage, Family, and the State in Contemporary China.” Pacific Affairs 57, no. 2: 213–236.

[cars70043-bib-0090] World Bank . 2022. China: Gender Data Portal . World Bank. Retrieved from: https://genderdata.worldbank.org/countries/china/#:~:text=In%20China%2C%20the%20labor%20force,labor%20force%20participation%20has%20decreased.

[cars70043-bib-0091] Wu, A. X. , and Y. Dong . 2019. “What Is Made‐in‐China Feminism (s)? Gender Discontent and Class Friction in Post‐Socialist China.” Critical Asian Studies 51, no. 4: 471–492.

[cars70043-bib-0092] Wu, Y. , and D. Qi . 2017. “A Gender‐Based Analysis of Multidimensional Poverty in China.” Asian Journal of Women's Studies 23, no. 1: 66–88.

[cars70043-bib-0093] Yang, S. , and A. Li . 2009. “Legal Protection Against Gender Discrimination in the Workplace in China.” Gender & Development 17, no. 2: 295–308.

[cars70043-bib-0094] Yodanis, C. L. 2002. “Producing Social Class Representations: Women's Work in a Rural Town.” Gender & Society 16, no. 3: 323–344.

[cars70043-bib-0095] Yucel, D. , and H. Chung . 2023. “Working From Home, Work–Family Conflict, and the Role of Gender and Gender Role Attitudes.” Community, Work & Family 26, no. 2: 190–221.

[cars70043-bib-0096] Zhong, S. , M. Wang , Y. Zhu , Z. Chen , and X. Huang . 2022. “Urban Expansion and the Urban–Rural Income Gap: Empirical Evidence From China.” Cities 129: 103831.

